# Biomarkers of reactive resistance and early disease progression during chemotherapy plus bevacizumab treatment for colorectal carcinoma

**DOI:** 10.18632/oncotarget.1811

**Published:** 2014-03-22

**Authors:** Hidetoshi Hayashi, Tokuzo Arao, Kazuko Matsumoto, Hideharu Kimura, Yosuke Togashi, Yoshinori Hirashima, Yosuke Horita, Satoru Iwasa, Natsuko Tsuda Okita, Yoshitaka Honma, Atsuo Takashima, Ken Kato, Tetsuya Hamaguchi, Yasuhiro Shimada, Kazuhiko Nakagawa, Kazuto Nishio, Yasuhide Yamada

**Affiliations:** ^1^ Department of Genome Biology, Kinki University Faculty of Medicine, Osakasayama City, Osaka, Japan; ^2^ Department of Medical Oncology, Kinki University Faculty of Medicine, Osakasayama City, Osaka, Japan; ^3^ Department of Medical Oncology, Kishiwada Municipal Hospital, Kishiwada, Osaka, Japan; ^4^ Gastrointestinal Medical Oncology Division, National Cancer Center Hospital, Chuo-ku, Tokyo, Japan; ^5^ Department of Medical Oncology, Oita University, Hazama-cho, Yufu, Oita, Japan; ^6^ Department of Chemotherapy, Toyama Prefectural Central Hospital, Toyama, Toyama, Japan

**Keywords:** placental growth factor (PlGF), vascular endothelial growth factor (VEGF), colorectal carcinoma, bevacizumab, angiogenesis

## Abstract

Molecular markers for predicting or monitoring the efficacy of bevacizumab in patients with metastatic colorectal cancer (mCRC) remain to be identified. We have now measured the serum concentrations of 25 angiogenesis-related molecules with antibody suspension bead array systems for 25 mCRC patients both before and during treatment in a previously reported phase II trial of FOLFIRI chemotherapy plus bevacizumab. The serum concentration of vascular endothelial growth factor–A (VEGF-A) decreased after the onset of treatment (P < 0.0001), whereas that of placental growth factor increased (P < 0.0001). Significant differences in the levels of several factors (such as VEGF-A, soluble VEGF receptor–2, and interleukin-8) were apparent between responders and nonresponders during treatment. The rapid and pronounced decrease in serum VEGF-A level after treatment onset was apparent in all subjects and was independent of the baseline concentration. However, four of nine nonresponders showed a subsequent early increase in the serum VEGF-A level. Our results thus suggest that an early increase in the serum VEGF-A concentration after the initial decrease is a potential predictive marker of a poor response and reactive resistance to bevacizumab plus chemotherapy.

## INTRODUCTION

Angiogenesis, defined as the formation of new blood vessels from a preexisting vasculature, is essential for tumor growth and the spread of metastases [1, 2]. Inhibition of angiogenesis is therefore considered a promising strategy for cancer treatment, with clinical application of this strategy being pursued in the form of multiple modalities that include the development of specific inhibitors of signaling by vascular endothelial growth factor (VEGF) and its cognate receptors (VEGFRs). Bevacizumab is a humanized monoclonal antibody specific for VEGF-A, a key inducer of angiogenesis in tumors, and it has been found to manifest clinical activity in patients with metastatic colorectal cancer (mCRC) [3]. Furthermore, the cytotoxicity of chemotherapy is blunted by the production of VEGF and other proangiogenic factors that recruit new endothelial cells and protect them from chemotherapy, and bevacizumab transiently “normalizes” the abnormal structure and function of the tumor vasculature to render it more efficient for oxygen and drug delivery [4]. Indeed, bevacizumab is effective against metastatic colorectal cancer (mCRC) mainly in combination with chemotherapeutic drugs.

The efficacy of chemotherapy plus bevacizumab varies among patients, however, and so the ability to identify tumors likely to be most sensitive to such treatment would help to optimize the implementation of this approach as well as provide important insight into the mechanisms of resistance. The identification of a biomarker predictive of bevacizumab treatment outcome has proven to be challenging. Angiogenesis is a complex and highly adaptive biological process, with multiple factors in addition to VEGF-A playing an essential role, including placental growth factor (PlGF), fibroblast growth factors (FGFs), platelet-derived growth factor (PDGF), angiopoietins, and various additional cytokines [5]. Reactive resistance to bevacizumab in combination with chemotherapy is mediated in part by hypoxia-inducible factor–1 (HIF-1) and its transcriptional activation of genes for multiple factors including VEGF-A and FGFs.

Extensive biomarker analysis has been conducted in numerous clinical trials of bevacizumab, with evaluation of the relation between circulating VEGF-A levels at baseline and treatment outcome having been performed in most cases [6]. Although a few studies have detected a significant correlation between the baseline serum concentration of VEGF-A and the outcome of antiangiogenic therapy [7], many others have not. The inconsistency of these results emphasizes the need for evaluation of predictive biomarkers in a dynamic manner—that is, before and after the onset of antiangiogenic treatment.

We have previously described a phase II study (AVASIRI trial) designed to investigate the efficacy of a bevacizumab plus FOLFIRI (folinic acid, 5-fluorouracil, irinotecan) regimen as a second-line treatment for individuals with metastatic colorectal cancer (mCRC) [8]. Promising results were obtained with regard to response rate (32%), progression-free survival (PFS) time (median of 11.6 months), and overall survival (OS) time (median of 21.4 months). Serum samples were collected at various time points during the trial for measurement of the levels of 25 angiogenesis-related molecules. We now present the results of the analysis of these serum samples from the AVASIRI trial.

## RESULTS

### Patient characteristics

Serum samples were available for all 25 patients treated with FOLFIRI and bevacizumab. The characteristics of the study patients are shown in Table 1. The median age was 62 years (range, 38–73), and the male/female distribution was 20/5. The overall response rate was 32%, with 8 patients showing a partial response, 15 stable disease, and 2 disease progression. Median progression-free survival (PFS) and overall survival (OS) were 11.6 months [95% confidence interval (CI), 6.9–16.4] and 21.4 months (95% CI, 12.0–30.8), respectively.

**Table 1:** Summary of patient characteristics and AVASIRI trial results

Median (range) age of patients (years)	62 (38–73)
ECOG performance status 0/1	16/9
Male/female	20/5
Primary lesion in colon/rectum	12/13
Prior treatment with/without FOLFOX	16/9
Overall response rate (%)	32 (90% CI, 17.0–50.4)
Median PFS (days)	349 (95% CI, 207–491)
Median OS (days)	642 (95% CI, 359–925)

Abbreviations not defined in text: ECOG, Eastern Cooperative Oncology Group; FOLFOX, folinic acid plus 5-fluorouracil plus oxaliplatin.

### Circulating levels of angiogenesis-related molecules before and during treatment with FOLFIRI and bevacizumab

**Figure 1:**
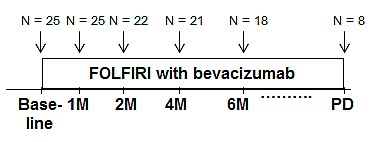
Flow diagram for analysis of the study subjects Paired serum samples were available for 25 patients at baseline and at 1 month after the onset of treatment, for 22 patients at 2 months, for 21 patients at 4 months, for 18 patients at 6 months, and for 8 patients at the onset of progressive disease (PD) or last follow-up.

**Figure 2:**
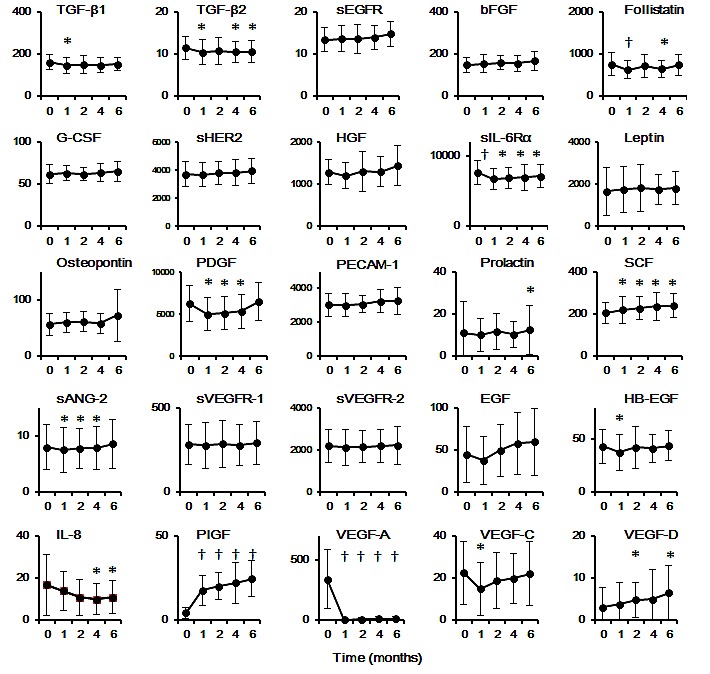
Serum concentrations of 25 angiogenesis-related molecules at baseline and at 1, 2, 4, and 6 months after the onset of FOLFIRI with bevacizumab treatment Data are means ± SD for the numbers of samples indicated in Figure 1. *P < 0.05, †*P* < 0.0001 versus the corresponding baseline value (Student's paired *t* test). All values represent picograms per milliliter.

We examined changes in the serum concentrations of 25 angiogenesis-related molecules between before (baseline) and after the onset of treatment with FOLFIRI plus bevacizumab (Figure 1). The baseline serum concentrations varied widely among individuals, with the values for VEGF-A, for example, ranging from 13 to 907 pg/mL. Significant changes in the serum levels of various molecules were apparent at various time points during treatment compared with baseline (Figure 2). Of note, the serum concentration of VEGF-A decreased markedly after the onset of treatment (from 337.7 ± 244.4 pg/mL at baseline to 1.9 ± 5.0, 5.6 ± 12.6, 8.2 ± 17.5, and 7.3 ± 20.8 pg/mL at 1, 2, 4, and 6 months, respectively; *P* < 0.0001), whereas that of PlGF showed a pronounced increase (from 4.1 ± 3.4 pg/mL at baseline to 17.6 ± 9.0, 19.9 ± 7.9, 21.9 ± 12.3, and 24.4 ± 10.8 pg/mL at 1, 2, 4, and 6 months, respectively; *P* < 0.0001). Given that these results were obtained with paired samples from the same individuals at baseline and after the onset of treatment, the observed changes were likely attributable to the administration of FOLFIRI plus bevacizumab.

### Serum concentrations of angiogenesis-related molecules and PFS

We divided the patients into two groups on the basis of progression-free survival (PFS) time. Given that the median PFS for patients with mCRC treated with chemotherapy plus bevacizumab in the second-line setting was previously found to be ~7 months [9], we dichotomized our patient population according to a PFS of 7 months (responders, ≥7 months; nonresponders, <7 months). None of the 25 molecules examined served as a predictive marker on the basis of the baseline serum concentrations, whereas significant differences in the levels of various molecules [including soluble VEGFR-2 (sVEGFR-2), interleukin (IL)–8, VEGF-A, and VEGF-C] at various time points during treatment were apparent between responders and nonresponders (Table 2).

**Table 2:** Serum concentrations of 25 angiogenesis-related molecules at baseline and at the indicated times after the onset of treatment with FOLFIRI plus bevacizumab in responders and nonresponders Data are means ± SD. The P values for comparisons between responders (RES) and nonresponders (non-RES) were determined with Student's unpaired t test; those of <0.05 are shown in bold.

							Serum concentratIon (pglml)								
	Baseline (N = 25)			1 month (N = 25)			2 months (N = 22)			4 months (N = 21)			6 months (N = 18)		
	RES	non-RES	P value	RES	non-RES	P value	RES	non-RES	P value	RES	non-RES	P value	RES	non-RES	P value
TGF-β1	164 ± 33	149 ± 46	0.443	145 ± 39	138 ± 46	0.732	142 ± 32	159 ± 70	0.594	151 ± 35	121 ± 26	0.067	157 ± 29	123 ± 28	0.086
TGE-β2	12±3	12±3	0.818	10±3	10±3	0.961	11±4	12±2	0.379	11±3	10±1	0.362	11±3	9±1	0.176
sEGFR	13±3	14±4	0.838	13±3	14±4	0.571	13±3	15±4	0.241	13±3	16±3	0.190	14±3	18±2	**0.018**
bEGE	146±38	159±26	0.352	150±47	167±33	0.346	149±35	182±25	**0.031**	149±40	177±29	0.115	157±43	195±39	0.156
Follistatin	736 ± 310	766 ± 198	0.783	627 ± 257	603 ± 143	0.778	664 ± 206	860 ± v388	0.284	620 ± 202	745 ± 185	0.242	655 ± 227	997 ± 131	**0.004**
G-CSF	61±11	64±10	0.610	63±10	62±8	0.947	62±7	60±11	0.119	65±10	59±12	0.347	64±10	65±19	0.966
sHER2	3697 ±920	3914 ±811	0.580	3645 ±971	3906 ±599	0.443	3615 ±917	4207 ±306	**0.040**	3737 ±840	4148 ± 1011	0.443	3700 ±856	4686 ±429	**0.010**
HGF	1222 ±279	1470 ±303	0.093	1155 ±325	1332 ±289	0.216	1157 ±293	1660 ±674	0.131	1201 ±274	1599 ±422	0.103	1289 ±362	1921 ±573	0.110
slL-6Rα	7883±1714	6899±1465	0.183	7037±1562	5905±825	**0.035**	6981 ±1672	6097±700	0.104	7020±1616	6266±2505	0.555	7134±1715	6816±1467	0.727
Leptin	1608±1048	2038±1364	0.475	1704±1045	2119±1237	0.457	1765±912	2054±1592	0.690	1823±702	1625±805	0.640	1903±836	1379±455	0.133
Osteopontin	54 ± 21	59 ± 18	0.582	58 ± 20	61 ± 16	0.665	57 ± 17	67 ± 21	0.366	54 ± 12	67 ± 32	0.441	65 ± 31	95 ± 86	0.538
PDGF	6297 ± 2439	6488 ± 1321	0.812	4978 ± 2276	5095 ± 1460	0.884	4770 ± 2124	5874 ± 1753	0.246	5224 ± 2244	5372 ± 1825	0.885	6095 ± 2085	7582 ± 2702	0.365
PECAM-1	3034 ±749	3116 ±469	0.753	3018 ±746	3081 ±548	0.823	2959 ±544	3294 ±257	0.072	3211 ±785	3468 ±323	0.317	3097 ±760	3857 ±711	0.121
Prolactin	10±17	10 ± 5	0.964	9±9	11 ± 5	0.451	11 ±9	15±9	0.374	10±6	13 ± 8	0.450	8±4	26 ± 19	0.159
SCF	207 ± 55	210 ± 44	0.892	218 ± 62	235 ± 71	0.587	218 ± 55	249 ± 55	0.270	226 ± 55	276 ± 91	0.297	227 ± 61	279 ± 20	**0.016**
sANG-2	8±4	10±3	0.139	7±4	9±3	0.292	7±4	10±2	**0.030**	7±4	11±3	**0.027**	8±4	12±3	0.062
sVEGFR-1	289±139	289±67	0.997	282±166	268±67	0.774	261 ±133	358±148	0.199	264±123	324±120	0.366	275±140	344±68	0.201
sVEGFR-2	2128 ± 873	2388 ± 662	0.445	2067 ± 917	2338 ± 785	0.483	2001 ± 750	2567 ± 799	0.171	1995 ± 792	2794 ± 418	**0.012**	2004 ± 887	2936 ± 473	**0.020**
EGF	50±34	26±28	0.097	38±30	31±28	0.628	58±32	31±21	**0.038**	59±39	56±37	0.861	59±40	62±43	0.882
HB-EGF	44±14	42±24	0.897	39±13	38±26	0.966	40±13	48±32	0.573	43±14	35±9	0.138	46±15	35±12	0.183
IL-8	14±15	24±13	0.115	10±6	19±7	**0.027**	8±6	17±11	0.109	8±6	15±10	0.216	9±6	18±10	0.165
PIGE	4±3	5±4	0.635	17±9	20 ± 9	0.473	19±8	19±6	0.878	18±7	34 ± 18	0.127	22±8	33 ± 15	0.246
VEGF-A	333 ±295	351 ± 143	0.842	1 ±3	4±6	0.228	0 ± 1	20 ± 18	**0.046**	0 ± 1	33±22	**0.027**	0 ± 1	42 ± 38	0.198
VEGE-C	23±16	23±16	0.995	15±15	16±7	0.813	16±13	27±12	0.082	17±12	28±8	**0.047**	21±16	25±11	0.603
VEGF-D	3±3	1±2	0.235	4±4	1±2	0.083	5±4	4±6	0.626	5±8	6±5	0.713	6±6	7±8	0.765

### Relation between FOLFIRI-bevacizumab treatment and changes in serum VEGF-A level

**Figure 3:**
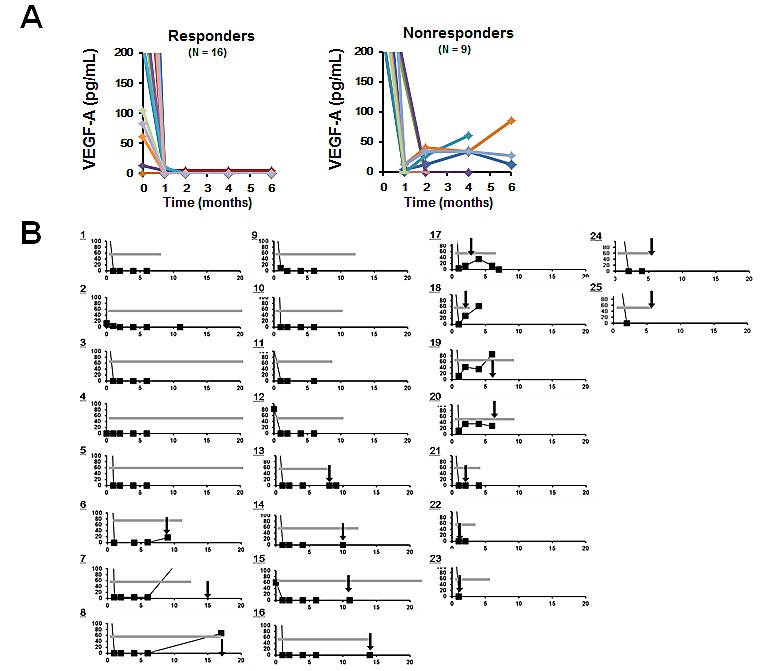
Analysis of changes in the serum concentration of VEGF-A (A) Time course of serum VEGF-A level in responders and nonresponders. (B) Time course of serum VEGF-A concentration in relation to the duration of treatment with bevacizumab plus chemotherapy (gray bars) and the detection of disease progression (black arrows). Cases 1 to 16 and 17 to 25 correspond to responders and nonresponders, respectively. The vertical and horizontal axes represent serum VEGF-A (pg/mL) and time (months), respectively.

Finally, we investigated the relation between changes in the serum concentration of VEGF-A and the duration of treatment with FOLFIRI plus bevacizumab (Figure 3). Several patients manifested an increase in the serum VEGF-A level around the time of disease progression relative to the lowered value apparent after the onset of treatment and during its administration for several months. Of note, four nonresponders showed an early increase in the serum concentration of VEGF-A (cases 17–20 in Figure 3B). The PFS of these four patients was significantly shorter than that of the other 21 patients (200 versus 373 days, respectively; *P* = 0.009, Student's unpaired *t* test), suggesting that an early increase in serum VEGF-A level subsequent to an initial decrease is predictive of early resistance to bevacizumab. On the other hand, the serum concentration of VEGF-A remained stable at the time of disease progression in other patients (cases 13–16). Patient 15 continued treatment with bevacizumab, in combination with a different chemotherapy regimen (mFOLFOX6), beyond disease progression.

## DISCUSSION

The introduction of novel molecularly targeted therapies, including antiangiogenic and anti–epidermal growth factor receptor (EGFR) agents, has increased the options available for treatment of mCRC [9]. At present, bevacizumab in combination with fluoropyrimidine-based chemotherapy is widely recognized as a standard treatment for mCRC [3, 9, 10]. However, no biomarker has previously been identified as a predictor of benefit from bevacizumab treatment, with the identification of such a molecular biomarker being a current priority of clinical research [11]. In the present study, we have addressed this issue by measuring the serum levels of multiple angiogenesis-related factors both before and during treatment of mCRC patients with bevacizumab plus FOLFIRI.

Most previous studies have found that the circulating concentration of VEGF-A, as measured with standard enzyme-linked immunosorbent assays, increases after the onset of antiangiogenic treatment [7, 12-18], whereas more recent studies have shown a decrease in VEGF-A levels after treatment onset [19-21]. We have now shown that treatment with bevacizumab plus chemotherapy was associated with a rapid and highly significant decrease in the serum concentration of VEGF-A that was independent of the baseline concentration and which, in most cases, remained apparent throughout the duration of therapy, similar to the results of a previous pharmacodynamic analysis of angiogenesis-related factors [22]. Although it remains unclear whether circulating VEGF-A in individuals treated with bevacizumab is free or bound to the antibody, given that bevacizumab is administered at doses high enough to give rise to such binding, our results suggest that the antibody suspension bead array system adopted in the present study measures VEGF-A that is free of bevacizumab.

An initial decrease in serum VEGF-A level was observed in all patients of the present study. However, some patients manifested a subsequent early small but definite increase in this parameter. This latter finding may be related to the assay measuring free VEGF-A and may therefore reflect a compensatory increase in the circulating concentration of this factor. Our observation that the PFS of such patients was shorter than that of the other subjects suggests that the development of acquired resistance to bevacizumab treatment may be driven in part by loss of the ability to suppress the circulating level of free VEGF-A. VEGF-A promotes the survival of and increases resistance to chemotherapy in cancer cells. Chemotherapy acts as an “accidental” antiangiogenic therapy (action), whereas VEGF-A and other proangiogenic factors recruit new endothelial cells and protect them from the cytotoxicity of chemotherapy (reaction) [4, 23]. Bevacizumab is thought to block this reaction. From this perspective, our results suggest that an early increase in VEGF-A levels after the initial decrease is a potential predictive marker of reactive resistance to bevacizumab that results in a shorter PFS in patients treated with the combination of FOLFIRI and bevacizumab.

Despite the predominant role of VEGF-A, multiple other factors contribute to regulation of the complex and highly adaptive process of angiogenesis. Investigation of potential biomarkers other than VEGF-A is thus important, given the role of these other factors in tumor angiogenesis and vessel maturation. However, only a few studies have previously examined multiple angiogenesis-related proteins during bevacizumab treatment in a dynamic manner [22]. We have now detected significant treatment-induced changes in the serum concentrations of several angiogenesis-related molecules including PlGF. Previous biomarker analyses also described an increase in the circulating concentration of PlGF in response to VEGF-targeted treatment [12, 14, 15, 17, 22]. Indeed, targeting of PlGF is under consideration as a novel approach to prevent tumor escape from VEGF-targeted therapy [24]. However, we did not detect a significant difference in serum PlGF levels after bevacizumab administration between responders and nonresponders in the present study. A previous study also found that the combination of antibodies to PlGF and those to VEGF-A did not yield a greater antitumor effect in vitro or in vivo compared with antibodies to VEGF-A alone [25]. Our data thus suggest that the increase in circulating PlGF level observed after the onset of bevacizumab treatment does not play a major role in the development of resistance to bevacizumab in the clinical setting.

On the other hand, we detected significantly higher serum concentrations of several angiogenesis-related factors [such as IL-8, soluble angiopoietin II (sANG-2), basic FGF (bFGF), stem cell factor (SCF), and VEGF-C] in nonresponders compared with responders at various time points during treatment. Resistance to VEGF-A pathway inhibitors might occur through VEGF-A–independent mechanisms, such as up-regulation of other proangiogenic factors [26-28]. Given that targeting of these molecules may provide a basis for novel approaches to prevent tumor escape from bevacizumab treatment, further analysis of multiple angiogenesis-related factors in a large number of patients is warranted.

In conclusion, our present results indicate that an early increase in the serum concentration of VEGF-A after the initial decrease may be a potential predictive marker of a poor response and reactive resistance to bevacizumab plus chemotherapy.

## METHODS

### Patients

The main inclusion criteria for the present study were the same as those previously described for the AVASIRI trial [8]. In brief, they comprised a histologically confirmed diagnosis of colorectal cancer; failure of first-line treatment with 5-fluorouracil– or oxaliplatin-based chemotherapy without bevacizumab or CPT-11 (irinotecan); measurable disease according to RECIST (ver. 1.0); and metastatic disease deemed unresectable at baseline. Enrolled patients received biweekly administrations of the FOLFIRI regimen, consisting of CPT-11 (150 mg/m^2^) on day 1, given as a 2-h infusion concurrent with leucovorin (folinic acid, 200 mg/m^2^), followed by 5-fluorouracil given by injection (400 mg/m^2^) and then as a 46-h continuous infusion (2400 mg/m^2^). Bevacizumab was administered at a biweekly dose of 10 mg/kg before the FOLFIRI regimen. Treatment was discontinued in the event of disease progression, unacceptable toxicity, or withdrawal of consent. Patients underwent a computed tomography scan after every four cycles of treatment for evaluation of tumor response. They provided written informed consent to receive the treatment and to participate in translational analyses.

### Sample collection and analysis

Blood samples were obtained from all assigned patients at baseline (before the first dose of study drugs) as well as at 1, 2, 4, and 6 months after the onset of the treatment protocol (Figure 1). In addition, blood samples from eight patients who received the study treatment for >6 months were obtained at the time of disease progression or last follow-up. Serum separated from the blood samples was stored at –80°C until analysis.

The serum levels of VEGF-A, VEGF-C, VEGF-D, PlGF, epidermal growth factor (EGF), IL-8, and heparin-binding EGF-like growth factor (HB-EGF) were measured with the use of a Milliplex MAP Human Angiogenesis/Growth Factor Magnetic Bead Panel (Merck Millipore, Billerica, MA, USA). Magnetic antibody-conjugated beads were subjected to ultrasonic treatment for 30 s and then to vortex-mixing for 1 min in order to reduce bead aggregation. All samples, quality controls, and standards were prepared as recommended with the supplied diluents and were processed in duplicate batches. Assay buffer (200 μL) was added to each well and then decanted. Each sample (25 μL) and the prepared beads (25 μL) were then added to the wells together with buffering solutions. The plate was sealed, incubated overnight at 4°C, and washed three times, after which detection antibodies (25 μL) were added to each well and the plate was incubated for 1 h at room temperature. Streptavidin-phycoerythrin (25 μL) was then added to each well, after which the plate was incubated for an additional 30 min at room temperature and washed three times. Sheath fluid (100 μL) was finally added to each well, and the assay plate was analyzed with the Luminex 100 instrument.

The serum levels of transforming growth factor (TGF)–β1 and TGF-β2 were measured with a Milliplex MAP Multi-Species TGFβ 3-Plex panel (Merck Millipore), whereas those of various additional factors related to angiogenesis were measured with a Bio-Plex Pro Human Cancer Biomarker Panel 1, 16-Plex (Bio-Rad, Hercules, CA, USA) as previously described [29]. The latter factors included soluble EGFR (sEGFR), bFGF, osteopontin, PDGF–AB/BB, follistatin, granulocyte colony-stimulating factor (G-CSF), platelet endothelial cell adhesion molecule–1 (PECAM-1), prolactin, soluble human EGF receptor 2/NEU (sHER2/NEU), hepatocyte growth factor (HGF), SCF, sANG-2, soluble IL-6 receptor α (sIL-6Rα), leptin, sVEGFR-1, and sVEGFR-2.

### Statistical analysis

Serum factor levels at baseline (pretreatment) were compared with those at 1, 2, 4, or 6 months after treatment onset with the use of Student's paired *t* test in order to evaluate the significance of changes induced by the study treatment. The relations between treatment efficacy and serum factor levels were analyzed with Student's unpaired *t* test. A *P* value of <0.05 was considered statistically significant. All statistical tests were performed with SPSS version 14.0 software (SPSS, Chicago, IL, USA).
